# Early Outcomes of Low Postoperative Bleeding after Off-Pump Coronary Artery Bypass Grafting

**DOI:** 10.21470/1678-9741-2018-0341

**Published:** 2019

**Authors:** Weitie Wang, Yong Wang, Hulin Piao, Bo Li, Tiance Wang, Dan Li, Zhicheng Zhu, Rihao Xu, Kexiang Liu

**Affiliations:** 1 Department of Cardiovascular Surgery, 2nd Hospital of Bethune, Jilin University, Changchun, Jilin, China.

**Keywords:** Off-Pump Coronary Artery Bypass, Respiration, Artificial, Coronary Artery Disease, Reoperation, Hemodynamics

## Abstract

**Objective:**

To investigate whether low bleeding influences the early outcomes after off-pump coronary artery bypass grafting (CABG).

**Methods:**

Retrospective analysis of ischemic heart disease patients who underwent off-pump CABG from January 2013 to December 2017. Patients were divided into low-bleeding group (n=659) and bleeding group (n=270), according to total drainage from chest tube during the first postoperative 12 hours. Clinical material and early outcomes were compared between the groups.

**Results:**

Baseline was similar in the two groups. Operation time was 270±51 min in the low-bleeding group and 235±46 min in the bleeding group (*P*<0.0001). The low-bleeding group presented smaller drainage during the first 12 h (237±47 ml) and shorter mechanical ventilation time (6.86±3.78 h) than the bleeding group (557±169 ml and 10.66±5.19 h, respectively) (*P*<0.0001). Hemodynamic status was more stable in the low-bleeding group (*P*<0.0001) and usage rate of more than two vasoactive agents in this group was lower than in the bleeding group (*P*<0.0001). Number of distal anastomosis, reoperation for bleeding, suddenly increase in chest tube output, intensive care unit (ICU) stay, hospital stay, and other early outcomes had no statistical significance between the groups (*P*>0.05).

**Conclusion:**

Postoperative bleeding < 300 ml/12 h in off-pump CABG patients did not require blood product transfusion and reoperation and that would contribute to reduction in mechanical ventilation time and maintaining hemodynamic stability. Bleeding < 800 ml during the first postoperative 12 h did not increase infection rates and ICU length of stay.

**Table:** 

Abbreviations, acronyms & symbols				
AKI	= Acute kidney injury		LCOS	= Low cardiac output syndrome
BMI	= Body mass index		LIMA	= Left internal mammary artery
CABG	= Coronary artery bypass grafting		LVEF	= Left ventricular ejection fraction
CAD	= Coronary artery disease		MI	= Myocardial infarction
COPD	= Chronic obstructive pulmonary disease		NYHA	= New York Heart Association
CPB	= Cardiopulmonary bypass		PCI	= Percutaneous coronary intervention
CRD	= Chronic renal dysfunction		RBC	= Red blood cell
CRF	= Chronic renal failure		RITA	= Right internal thoracic artery
DM	= Diabetes mellitus		SD	= Standard deviation
ECG	= Electrocardiogram		SPSS	= Statistical Package for the Social Sciences
GFR	= Glomerular filtration rate		SVG	= Saphenous vein graft
IABP	= Intra-aortic balloon pump		UDPB	= Universal definition of perioperative bleeding
ICU	= Intensive care unit			

## INTRODUCTION

With aspirin being recommended to be administered to coronary artery bypass grafting (CABG) patients preoperatively since 2010, the risk of cardiovascular events significantly decreased^[1-3]^. However, it also increased hemorrhage-related risks, surgical re-explorations, the volume of red blood cell (RBC) transfusions^[4]^, and in-hospital mortality^[5,6]^. Numerous studies had reported the effects of massive bleeding, surgical re-explorations, or blood product transfusions on surgical mortality^[7]^. However, few studies investigated the effects of low postoperative bleeding on the hemodynamic status. Undoubtedly, less bleeding has some advantage, and it would avoid blood transfusions and decrease risk of reoperations. Besides, low postoperative bleeding after cardiac surgery had also shown to reduce the incidence of hemodynamic instability and to promote recovery after surgery. In order to evaluate the potential effects of low postoperative bleeding in patients undergoing off-pump CABG, we performed this retrospective cohort study to analyze the association between low postoperative bleeding and perioperative outcomes.

## METHODS

This is a retrospective study and it was approved by the Ethics Committee of the Second Hospital of Jilin University. Consent form was obtained from every patient before discharge. All operations were performed by the same surgeon (K.L.). A total of 1052 patients underwent surgical revascularization from January 2013 to December 2017. Inclusion criteria were: 1) patients with ischemic heart disease who met surgical revascularization criterion; 2) off-pump CABG without blood product transfusions and postoperative reoperations for bleeding; 3) no other cardiac diseases which required concomitant interventions, such as ventricular septal defect, medium to severe mitral regurgitation, and left ventricle aneurysm; 4) patients who regularly took acetylsalicylic acid until operation^[8-10]^; 5) patients without other system diseases, especially hematological diseases; and 6) chest tube drainage during the first 12 h less than 800 ml (insignificant and mild), according to the universal definition of perioperative bleeding (UDPB) categories in adult cardiac surgery^[11]^. Therefore, a total of 929 patients were enrolled in this study. All patients were further divided into two subgroups according to their postoperative bleeding volume: low-bleeding group (<300 ml/12 h) and bleeding group (300-800 ml/12 h). All patients were followed up until the 6th postoperative month after discharge.

### Surgical Procedures

All surgery was performed through a median sternotomy. Left/right internal mammary artery and saphenous vein grafts (SVG) were harvested at the same time using “no-touch” technique. Deep pericardial sutures were performed after incision in the pericardium. Heparin was administered (1 mg/kg). The Medtronic Octopus apical suction positioning device and Starfish apical suction positioning device (Medtronic, Inc., Minneapolis, Minnesota, USA) were used for stabilization. Surgical revascularization was always started from the left internal mammary artery (LIMA) to the left anterior descending coronary territory. Then, sequential technique was employed to the right coronary artery, left circumflex and diagonal artery by one SVG. The quality of the anastomosis was assessed by transit-time flow probe (Medistim Butterfly Flow Meter, Oslo, Norway). All the target vessels were exposed and controlled with silastic sling. A CO_2_-blower mister device was utilized to achieve the visualization of the operative field. After anastomosis, heparin was neutralized with 50 mg of protamine and 1 g of calcium gluconate. A cell salvage device was used in all surgeries and the salvaged blood was reinfused into the patient during the operation. To avoid hypothermia-induced arrhythmia, central temperature was maintained above 36 ºC.

Baseline clinical data included age, sex, body mass index, smoking, New York Heart Association (NYHA) class, previous percutaneous coronary intervention (PCI), diabetes mellitus (DM), chronic renal dysfunction (CRD), previous myocardial infarction (MI), recent MI, congestive heart failure, hypertension, hyperkinemia, chronic obstructive pulmonary disease (COPD), stroke, prior cerebrovascular accident, abnormal motion of the segmental cardiac wall, left ventricular ejection fraction (LVEF), anatomical severity of coronary artery disease (CAD), hemoglobin, PO_2_, activated partial thromboplastin time, international normalized ratio, partial thrombin time, thrombin time, fibrinogen, and EuroSCORE. Operative data included operation time, number of distal anastomosis, the use of SVG, LIMA and right internal thoracic artery (RITA), the use of composite graft, and prophylactic intra-aortic balloon pump (IABP) support. Postoperative data included surgical mortality, drainage during the first 12 h, re-explorations for bleeding, duration of mechanical ventilation, prolonged postoperative ventilatory support (longer than 24 hours), the lengths of intensive care unit (ICU) stay and hospital stay, ventricular arrhythmia, low cardiac output syndrome (LCOS), sudden increase in chest tube output, tamponade, stroke, MI, hemodynamic instabilities (systolic pressure < 90 mmHg and heart rate > 120 bpm), and application of more than two vasoactive agents. Bivariate analyses were used to examine differences in baseline characteristics between the two groups.

The primary end point of this study was overall death, including in-hospital mortality and death occurring within 30 days after surgery. The secondary end points were major postoperative morbidities, such as LCOS, new onset of acute MI, and other cardiac-related complications. Follow-up information was obtained by outpatient clinic visit or telephone calls. All patients underwent echocardiographic reexamination at the 6^th^ postoperative month.

### Definitions

Surgical mortality was defined as in-hospital death or death occurring within 30 days of the surgery. The secondary end points were other operation-related complications. Resternotomy for bleeding was defined as reoperation to control bleeding within 36 hours following the initial surgery. Postoperative MI was defined by the appearance of new Q waves in two or more contiguous leads on the electrocardiogram (ECG). Postoperative LCOS was defined as the requirement for IABP and/or inotropic support for longer than 30 mins. Postoperative atrial or ventricular arrhythmia was defined as any episode of atrial/ventricular fibrillation, which was recognized by the monitoring system on a rhythm strip or the 12-lead ECG. Postoperative respiratory failure was defined as duration of mechanical ventilation longer than 72 hours or in-hospital re-intubation. Postoperative pneumonia was a positive result in a sputum culture requiring anti-infective treatment or chest X-ray diagnosis of pneumonia following cardiac surgery. Stroke was defined as new onset of permanent neurological events lasting over 24 hours. Deep sternal wound infection was bone-related, any drainage of purulent secretions from the sternotomy wound, and instability of the sternum. Acute kidney injury (AKI) was defined and classified according to the criteria proposed by the Acute Kidney Injury Network. Chronic renal failure (CRF) was diagnosed in patients whose glomerular filtration rate (GFR) declined to 15-20 ml/minute, with severe symptoms related to uraemia, requiring renal replacement therapy. Blood loss (measured by chest tube drainage) was recorded at or close to 12 h after surgery. More than two vasoactive agents indicated that dopamine and glyceryl trinitrate were routinely used during and after operation, while adrenaline or norepinephrine were added if the blood pressure was < 90 mmHg, despite dopamine and glyceryl trinitrate infusions.

### Statistics

Continuous data were expressed as mean ± standard deviation (SD), categorical variables were expressed as numbers (percentages). Normally and non-normally distributed continuous variables were compared using Student’s t-test and Mann-Whitney U test, respectively. The Fisher’s exact test or the chi-square test was used to compare categorical variables. *P*-value < 0.05 was considered statistically significant. All statistical analyses were carried out by the Statistical Package for the Social Sciences (SPSS) software (version 19.0).

## RESULTS

### Study Population

There were 659 patients in the low-bleeding group and 270 patients in the bleeding group. The mean age was 60.61±8.23 and 60.53±8.32 years in the low-bleeding group and the bleeding group, respectively. There were no significant differences in hemoglobin, PO_2_, activated partial thromboplastin time, international normalized ratio, partial thrombin time, thrombin time, and fibrinogen. More details of the baseline characteristics were shown in Table 1. There were no significant differences in age, gender, obesity, smoking, NYHA class III-IV, previous MI, previous PCI, hypertension, DM, CRF, recent MI, congestive heart failure, hyperlipemia, COPD, prior cerebrovascular accident, abnormal motion of the segmental cardiac wall, LVEF, and EuroSCORE between the two groups.

**Table 1:** Patients' baseline and procedural characteristics after matching.

	Low-bleeding group (N=659)	Bleeding group (N=270)	*P*-value
Age (years old)	60.61±8.23	60.53±8.32	0.8934
Males	425 (64.49%)	178 (65.93%)	0.6775
Obesity (BMI > 30 kg/m^2^)	345 (52.35%)	142 (52.59%)	0.9469
Smoking	311 (47.19%)	125 (46.30%)	0.8037
NYHA class III-IV	405 (61.46%)	162 (60.00%)	0.6793
Previous myocardial infarction (MI)	331 (50.23%)	142 (52.59%)	0.5127
Previous PCI	67 (10.17%)	28 (10.37%)	0.9260
Hypertension	336 (50.99%)	141 (48.51%)	0.7322
Diabetes mellitus	216 (32.78%)	92 (34.07%)	0.7030
Chronic renal dysfunction	0	0	-
Recent MI	0	0	-
Congestive heart failure	54 (8.19%)	18 (6.67%)	0.4292
Hyperlipemia	435 (66.01%)	177 (65.56%)	0.8947
COPD	45 (6.83%)	19 (7.04%)	0.9093
Prior cerebrovascular accident	188 (28.53%)	69 (25.56%)	0.4698
Abnormal motion of the segmental cardiac wall	364 (55.24%)	138 (51.11%)	0.2521
LVEF	56.91±4.49	56.41±4.59	0.1261
Extent of CAD			
Left main stem disease	191 (28.98%)	78 (28.89%)	0.9770
Three vessels	580 (88.01%)	241 (89.26%)	0.5903
Two vessels	43 (6.52%)	17 (6.30%)	0.8975
Hemoglobin (mg/l)	129.27±18.81	128.99±19.01	0.8373
PO_2_	83.21±11.23	82.19±12.44	0.2237
Activated partial thromboplastin time (s)	11.18±2.24	11.19±2.33	0.9513
International normalized ratio	0.99±0.12	0.98±0.11	0.2379
Partial thrombin time (s)	35.12±8.68	35.11±8.71	0.9873
Thrombin time (s)	14.33±3.57	14.29±3.54	0.8765
Fibrinogen (g/l)	3.68±0.99	3.69±1.01	0.8895
Logistic EuroSCORE	6.12±2.01	6.08±1.97	0.7818

BMI=body mass index; CAD=coronary artery disease; COPD=chronic obstructive pulmonary disease; LVEF=left ventricular ejection fraction; NYHA=New York Heart Association; PCI=percutaneous coronary intervention

### Intra-operative Data

The operation time was 270±51 min in the low-bleeding group and 235±46 min in the bleeding group (*P*<0.0001). The number of distal anastomosis (ranging from two to five) was 3.37±0.79 in the low-bleeding group and 3.36±0.80 in the bleeding group. Internal mammary artery was used in all patients, while the number of SVG was 657 in the low-bleeding group and 269 in the bleeding group. Postoperative hemoglobin was 111.42±21.56 and 112.31±22.07 in the low-bleeding group and the bleeding group, respectively. Prophylactic IABP support was used in four (0.61%) patients in the low-bleeding group and in three (1.11%) in the bleeding group (*P*=0.4198). The details are shown in Table 2.

**Table 2:** Perioperative characteristics after matching.

	Low-bleeding group (N=659)	Bleeding group (N=270)	*P*-value
Operation time (min)	270±51	235±46	<0.0001
No. of distal anastomosis	3.37±0.79	3.36±0.80	0.8615
SVG use	657(99.70%)	269(99.62%)	0.8704
LIMA use	657(99.70%)	269(99.62%)	0.8704
RITA use	2(0.30%)	1(0.38%)	0.4353
Composite grafting	657(99.70%)	269(99.62%)	0.8704
Hemoglobin after operation (mg/l)	111.42±21.56	112.31±22.07	0.5706
Prophylactic IABP support	4(0.61%)	3(1.11%)	0.4198

IABP=intra-aortic balloon pump; LIMA=left internal mammary artery; RITA=right internal thoracic artery; SVG=saphenous vein graft

**Fig. 1:**
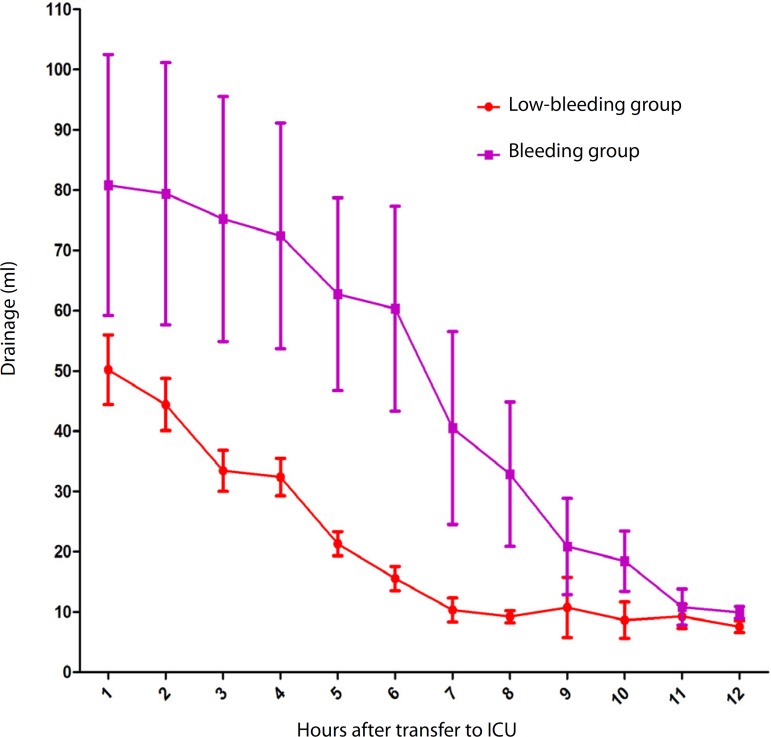
Patient’s bleeding of every hour after transfer to intensive care unit (ICU).

**Fig. 2:**
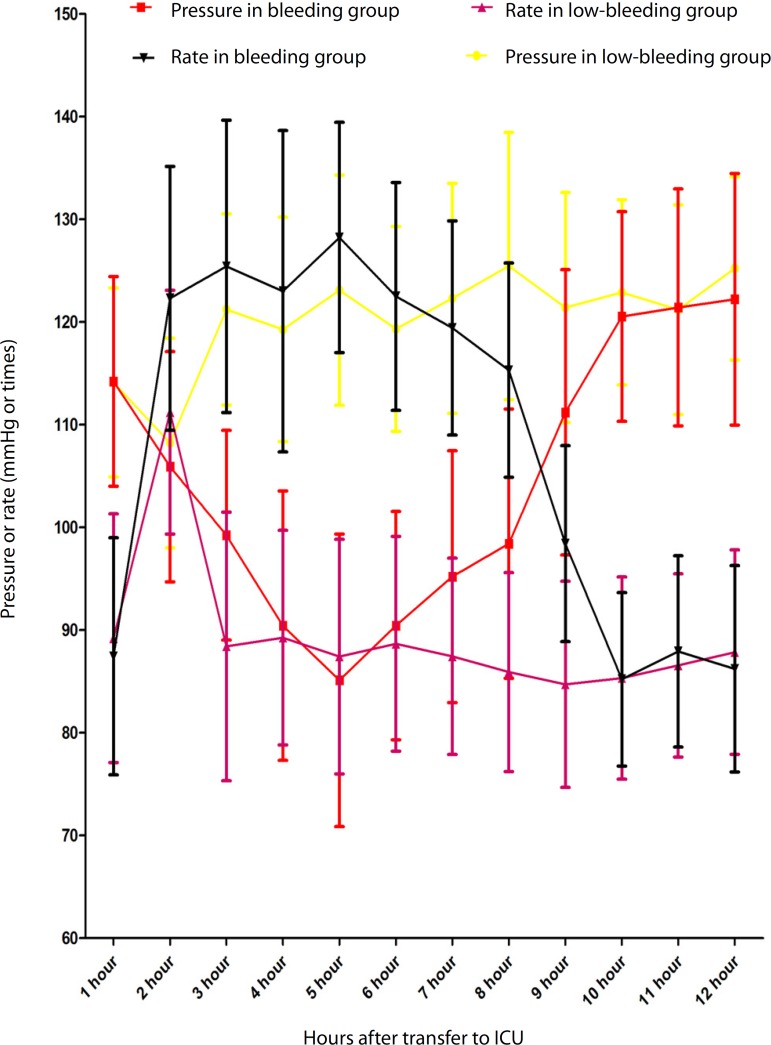
Hemodynamic status including systolic pressure and heart rate. Extubation happened when the systolic pressure and heart rate were simultaneously stable. ICU = intensive care unit

Drainage (Figure 1) during the first 12 h in the low-bleeding group (237±47 ml) was significantly lower than in the bleeding group (557±169 ml) (*P*<0.0001). Time of mechanical ventilation (6.86±3.78 h) in the low-bleeding group was also significantly shorter than in the bleeding group (10.66±5.19 h). Hemodynamic status (Figure 2) (including systolic pressure and heart rate) was more stable in the low-bleeding group (*P*<0.0001) and the usage rate of more than two vasoactive agents in the low-bleeding group was also lower than in the bleeding group (*P*<0.0001). There were no significant differences in resternotomy for bleeding, sudden increase in chest tube output, ICU stay, hospital stay, hemoglobin, ventricular arrhythmia, LCOS, MI, prolonged postoperative ventilatory support longer than 24 hours, respiratory failure, pneumonia, tamponade, deep surgical sternal wound, hemoglobin at the first postoperative 12 hours, and LVEF before discharge. More details of the postoperative data were shown in Table 3. All discharged patients were followed up until the 6th postoperative month, with no death occurring within six months in the two groups.

**Table 3:** Postoperative data.

	Low-bleeding group (N=659)	Bleeding group (N=270)	*P*-value
*In-hospital*			
Surgical mortality	4(0.61 %)	2(0.74 %)	0.817
Resternotomy for bleeding	0	0	-
Duration of mechanical ventilation (hour)	6.86±3.78	10.66±5.19	<0.0001
Prolonged postoperative ventilatory support > 24 hours	7(1.06%)	3(1.11%)	0.948
ICU stay (days)	2.49±0.37	2.55±0.54	0.052
Hospital stay (days)	8.45±0.41	8.47±0.39	0.494
Ventricular arrhythmia	5 (0.76 %)	2(0.74 %)	0.977
Low output syndrome	3 (0.46 %)	1(0.37 %)	0.858
Drainage during the first 12 h (ml)	237±47	557±169	<0.0001
Sudden increase in chest tube output	0	0	-
Tamponade	0	0	-
Hemoglobin at the first 12 h after operation	103.25±18.89	102.97±17.79	0.835
PO_2_	119.22±21.56	118.89±22.71	0.835
Stroke	3 (0.46 %)	1(0.37 %)	0.858
Myocardial infarction	5 (0.76 %)	2(0.74 %)	0.977
Urine of the first 12 h (ml)	2765±387	2801±399	0.202
Renal dysfunction	3 (0.46 %)	1(0.37 %)	0.858
*Hemodynamic status*			
Systolic pressure < 90 mmhg	85 (12.89 %)	82(30.37 %)	<0.0001
Sinus rhythm rate > 120	155 (23.52 %)	102(37.78 %)	<0.0001
More than two vasoactive agents	125 (18.97 %)	69(25.56 %)	0.025

## DISCUSSION

For patients with CAD, aspirin had been proved to reduce MI, stroke, and overall mortality^[12]^. In addition, the continuous usage of aspirin has been proved to be effective in protecting the graft from thrombosis^[13,14]^. So, it had been recommended to be administered preoperatively. However, the antithrombotic medications also increased bleeding risks, including the need for blood transfusions and surgical re-explorations for postoperative bleeding, during and after cardiac surgery^[15,16]^. Bleeding has always been a significant problem in cardiac surgery. Massive bleeding would lead to more blood transfusions and increase chances of re-explorations, which added the perioperative mortality risk. Even at some circumstances in which bleeding did not require blood transfusions or surgical re-explorations, it would still affect the patients’ postoperative hemodynamic status, which would result in blood pressure fluctuations and organ malperfusion. Indeed, every patient would experience a certain degree of postoperative bleeding, which was related to surgical damage to blood vessels and worsened by hemostatic mechanisms. The widely use of preoperative antithrombotic and anticoagulation medications was also an important factor, leading to impaired coagulant balance. In order to reduce postoperative bleeding, traditional practice had typically involved cessations of oral aspirin for up to 7-10 days before cardiac surgery in low-risk patients^[17]^. However, for some patients with severe CAD, the benefits of receiving aspirin outweigh the risk of postoperative bleeding. Therefore, more and more patients continued antiplatelet agents before a scheduled off-pump CABG. Another factor resulting in massive postoperative bleeding was hemostasis technology. Perfect anastomosis was easy for hemostasis and would contribute to less bleeding. Furthermore, it was affected by the worsened coagulant mechanisms. But the impaired hemostasis mechanisms preoperatively caused by using antithrombotic medication did not mean we could perform hemostasis careless. Instead, it called for higher requirements on hemostasis for these patients, which was an effective method to reduce bleeding after operation.

Normally, cardiac surgery patients experience postoperative bleeding of 400-700 ml approximately. Mortality was influenced by bleeding partly because it had negative effects on hemodynamics, blood product transfusions, or re-explorations. Transfusion of blood products had been reported to increase mortality and incidence of complications, such as renal failure and postoperative infections^[11,18]^. Although RBC transfusions could correct the anemia caused by surgical bleeding, transfusions still increased the complications, even if the patients received only one unit of blood^[19,20]^. Other studies reported that re-explorations would also increase in-hospital mortality and risk of renal failure, as well as prolong ventilatory support and increase resource utilization^[21,22]^. The relationship between postoperative bleeding and mortality was investigated through blood product transfusion or re-exploration, because of the shortage of a precise definition of bleeding. However, blood transfusions and re-explorations didn’t always happen, as sometimes low bleeding after operation would not require blood product transfusion or re-exploration. So, the effects of postoperative low bleeding haven’t been reported. Therefore, we aimed to evaluate the effects on the hemodynamic status and the early outcomes of low bleeding on patients with off-pump CABG, which required no blood product transfusion or re-explorations. To the authors’ knowledge, this is the first study that examines the association between low blood loss and clinical outcomes.

However, we also came across the question that at what cut-off point bleeding becomes clinically significant. It must be calculated with a precise definition of bleeding. According to the recent definition^[11]^ for perioperative bleeding, which used multiple clinically relevant parameters to create a simple five-class system (insignificant, mild, moderate, severe, or massive) to classify perioperative bleeding, we could take the bleeding as an outcome measure. That research showed that bleeding less than 800 ml would not require blood product transfusions or re-explorations, which meant that the influence of blood product transfusion or re-exploration on mortality could be avoided. Moreover, another study^[23]^ showed that there was no significance in outcomes with bleeding between 150-300 ml during the first 12 hours. So, for the off-pump CABG patients in our study, postoperative bleedings of 800 ml/12 h and 300 ml/12 h were adopted, with bleeding less than 300 ml as low-bleeding group and bleeding 300-800ml as bleeding group. To avoid the influence of cardiopulmonary bypass (CPB) on renal failure and survival rate, we only included off-pump CABG patients, without the CPB assist. So, in this study, blood transfusion was basically the only variable influencing postoperative outcomes.

In our study, the hemodynamic status was more stable in the low-bleeding group. During the first 12 hours, the incidence of low blood pressure in the low-bleeding group was less significant than in the bleeding group, especially when comparing systolic pressure < 90 mmHg. The low blood pressure may be caused by the blood loss, leading to the rise of the heart rate as a reflection of low blood pressure. Low blood pressure and fast heart rate must be dealt with supplementary volume and/or rise of the positive vasoactive agents or the addition of another positive vasoactive agent, such as adrenaline and/or norepinephrine. Our study presented that low bleeding in the chest tube drainage would contribute to the hemodynamic status and reduce the usage of positive vasoactive agents.

Another influence of low blood drainage was the duration of mechanical ventilation. The tube drainage in the first several hours always ranged between 50-100 ml and slowly decreased as the time went by (Figure 1). The extubation happened in the first several hours after the patient returned to ICU. So, the hemodynamic status in the first several hours was important for extubation. In the bleeding group, bleeding during the first several hours always increased the chances of low blood pressure and fast heart rate (Figure 2), the instable hemodynamic status would make the doctors to be more careful about the decision of extubation, and this was the possible reason to the longer duration of mechanical ventilation in the bleeding group than in the low-bleeding group. However, the hypovolemia caused by slow blood loss was easy to observe and treat, so the instable hemodynamic status always lasted for a relatively short period, and the visceral organs were not influenced by the blood pressure fluctuations because of their self-regulation.

The published study^[24]^ showed that major bleeding requiring RBC transfusion would increase infection rates and ICU length of stay. In our study, both groups didn’t require blood product transfusions, despite the bleeding group lost more blood than the low-bleeding group. So, our research also demonstrated that bleeding less than 800 ml during the first 12 h did not increase infection rates. The length of ICU stay and hospital stay between the two groups had no significant difference. The possible reason was that the postoperative bleeding was gradually reduced after 12 hours and that the drainage of the chest tube in every hour was between 5-10 ml, which had little influence on hemodynamic status.

### Limitations

This study presents several limitations. Firstly, it was a retrospective observational study based on a single center with a relatively small sample size, which may influence the generalizability of the results. Secondly, postoperative bleeding in this study only included the amount of the chest tube drainage. And the intraoperative blood loss was not included in this study, which may produce biased results. Thirdly, the influence of a longer operation time under anesthetic state of the low-bleeding group on postoperative hemodynamic stability and injury of viscera organs were not definite. Finally, the midterm and long-term clinical outcomes need further investigations.

## CONCLUSION

In summary, our study demonstrated that postoperative bleeding less than 300 ml/12 h in off-pump CABG patients did not require blood product transfusions and reoperations and that would benefit the extubation and hemodynamic stability. Besides, our study also showed that bleeding less than 800 ml during the first 12 h did not increase infection rates and length of ICU stay. Further research with larger sample is needed to validate these results.

**Table:** 

Authors' roles & responsibilities	
WW	Conceived the study, participated in its design and coordination, and helped to draft the manuscript; final approval of the version to be published
YW	Conceived the study, participated in its design and coordination, and helped to draft the manuscript; final approval of the version to be published
HP	Conceived the study, participated in its design and coordination, and helped to draft the manuscript; final approval of the version to be published
BL	Conceived the study, participated in its design and coordination, and helped to draft the manuscript; final approval of the version to be published
TW	Conceived the study, participated in its design and coordination, and helped to draft the manuscript; final approval of the version to be published
DL	Conceived the study, participated in its design and coordination, and helped to draft the manuscript; final approval of the version to be published
ZZ	Conceived the study, participated in its design and coordination, and helped to draft the manuscript; final approval of the version to be published
RX	Conceived the study, participated in its design and coordination, and helped to draft the manuscript; final approval of the version to be published
KL	Conceived the study, participated in its design and coordination, and helped to draft the manuscript; final approval of the version to be published
